# The Antioxidant Effect of Selenium Is Enhanced by Cortisol Through Nrf2 Pathway in Bovine Endometrial Epithelial Cells

**DOI:** 10.3390/ani15081075

**Published:** 2025-04-08

**Authors:** Luying Cui, Jingyi Zhong, Jiangyao Duan, Wanting Li, Peng Mao, Junsheng Dong, Kangjun Liu, Long Guo, Heng Wang, Jianji Li

**Affiliations:** 1 College of Veterinary Medicine, Yangzhou University, Jiangsu Co-Innovation Center for Prevention and Control of Important Animal Infectious Disease and Zoonoses, Yangzhou 225009, China; lycui@yzu.edu.cn (L.C.); mx120221020@stu.yzu.edu.cn (J.Z.); mx120221010@stu.yzu.edu.cn (J.D.); mz120221595@stu.yzu.edu.cn (W.L.); dx120220201@stu.yzu.edu.cn (P.M.); junsheng@yzu.edu.cn (J.D.); kangjunliu@yzu.edu.cn (K.L.); yzdxgl@yzu.edu.cn (L.G.); 2 International Research Laboratory of Prevention and Control of Important Animal Infectious Diseases and Zoonotic Diseases of Jiangsu Higher Education Institutions, Yangzhou University, Yangzhou 225009, China; 3 Joint International Research Laboratory of Agriculture and Agriproduct Safety of the Ministry of Education, Yangzhou 225009, China

**Keywords:** cortisol, selenium, oxidative stress, Nrf2 pathway, bovine endometrial epithelial cells

## Abstract

Background: In dairy cows, the stress-related cortisol level increases the susceptibility to postpartum uterine diseases. Oxidative stress is an important component of the disease process and causes morphological and functional alterations in the bovine endometrium. Selenium (Se) has an antioxidant property, and an appropriate Se supplementation is recommended to enhance bovine disease resistance. Methods and results: Here, we aimed to answer two questions: (1) how does cortisol affect the oxidative status of bovine endometrial cells; and (2) does Se supplementation protect cells from oxidative injury with a high cortisol condition? The oxidative stress of primary bovine endometrial epithelial cells (BEECs) was established by *Escherichia coli* lipopolysaccharide (LPS) stimulation, as marked by the increased oxidative markers and the suppressions of antioxidant indicators and Nrf2 signaling. In the absence of LPS, cortisol levels of 15 ng/mL showed a more significant antioxidative effect than cortisol levels of 5 and 30 ng/mL. In the presence of LPS, cortisol levels of 15 and 30 ng/mL elicited antioxidation, whereas 5 ng/mL of cortisol did not. Regardless of LPS stimulation, Se pretreatment of 1, 2, and 4 μM protected BEEC from oxidative stress, as evidenced by the decreased oxidative markers, increased antioxidant indices, and the activated Nrf2 signaling. With the presence of 30 ng/mL of cortisol, there was an enhanced Se antioxidant effect, which can be abolished by the block of cortisol receptor. Conclusions: Both cortisol and Se elicited antioxidant properties in BEEC through the Nrf2 pathway. In addition, the Se antioxidation was enhanced by cortisol.

## Simple Summary

Stress in dairy cows raises the endogenous cortisol levels, increasing the risk of postpartum uterine diseases. Oxidative stress is a key factor that alters the endometrial structure and function. Selenium (Se), with its antioxidant property, can boost the cows’ resistance to these diseases. Our study aimed to explore the impact of cortisol on the oxidative status in the bovine endometrial epithelial cells and the protective role of Se against oxidative injury in the cells exposed to cortisol. We found that cortisol showed an antioxidative effect. Se pretreatment protected cells from oxidative stress, and this protection was enhanced by cortisol. In addition, we found that both cortisol and Se activated the same pathway (NRF2), but probably through a different mechanism of action. This study helps reveal the protective mechanism of Se in dairy cows with stressed conditions.

## 1. Introduction

Postpartum uterine infection is a worldwide problem for the cattle industry due to its negative impact on reproductive performance [1,2]. During the peripartum period, dairy cows are exposed to multiple stressors, such as pregnancy stress, labor stress, lactation stress, and pain stress, all of which increase the endogenous cortisol concentration [3]. Elevated cortisol levels reduce the phagocytic capacity of polymorphonuclear neutrophils (PMNs) and impair the uterus’s ability to clear bacteria, thereby exacerbating postpartum uterine infections and contributing to reproductive disorders [4,5].

Selenium (Se) is an essential trace element. The normal range of Se concentration in the blood of cattle is 0.08–0.16 mg/L [6]. However, Se deficiency has been found worldwide due to its uneven distribution, and leads to Se insufficiency in local livestock and poultry [7]. Se deficiency is indicated when the plasma Se level of cows is lower than 0.08 mg/L [8]. Cows in peripartum and early lactation periods are more prone to Se deficiency because of the negative nutritional balance. Long-term exposure of ruminants to a certain degree of Se deficiency increases the risk of endometritis and delays uterine involution [9,10]. An appropriate Se supplementation has been indicated to decrease the occurrence of reproductive disorders [10,11]. Administering a Se yeast supplement to cows during the late stages of gestation has been shown to improve their postpartum disease resistance [12].

Se exerts antioxidant functions mainly by binding to selenoproteins in the form of selenocysteine residues in vivo. Under normal circumstances, there is a dynamic balance between the antioxidant capacity and the production of oxygen free radicals in the body. Disruption of the balance gives rise to the accumulations of reactive oxygen species (ROS), reactive nitrogen (NO) free radicals, and the end product of lipid peroxide such as malondialdehyde (MDA), causing oxidative damage to cells and tissues, and producing a large amount of lactate dehydrogenase (LDH) [13]. Glutathione peroxidase (GSH-Px) and thioredoxin reductase (TRXR) are typical selenoproteins with antioxidant properties. For example, PMN kills the ingested bacteria by forming a highly oxidized intracellular environment, where GSH-Px helps protect cells from the oxidative damage caused by ROS metabolites [14]. An appropriate increase in plasma Se levels is positively correlated with the selenoprotein activity, and the activity of antioxidant enzymes, including GSH-Px, TRXR, catalase (CAT), and superoxide dismutase (SOD), and the total antioxidant capacity (T-AOC) [15]. Se supplementation has been proved to enhance the antioxidant function and alleviate the oxidative stress of cows in vivo [16] and in vitro [17].

The redox-sensitive transcription factor, nuclear factor erythroid 2-related factor 2 (Nrf2), can facilitate the transcription of various genes that codify the synthesis of antioxidant enzymes [18]. The Nrf2/HO-1 signaling pathway is now recognized as the principal protective response to oxidative stress [19,20]. It has been found that Se treatment protected bovine mammary epithelial cells (BMECs) through Nrf2 activation, which increased the expression and activity of TRXR and the antioxidative function [21]. Selenomethionine has been reported to promote the expression of Nrf2 pathway-related factors at both mRNA and protein levels in the bovine endometrial cell line evoked by a lipopolysaccharide (LPS) challenge [17]. We also observed the activation of Nrf2 and the enhanced antioxidant status of primary bovine endometrial stromal cells (BESCs) treated with sodium selenite [22].

Oxidative stress is a key component that leads to the disturbed uterine defense against pathogen invasion in stressed cows during the periparturient period [23]. A study has shown that cortisol regulated SOD and CAT, and played a positive role in oxidative defense in the hepatopancreas of marine molluscs [24]. However, the elevated glucocorticoid concentration in tissue has been reported to impair the Nrf2-dependent antioxidant response of mice liver [25,26]. These studies indicated that the effects of cortisol vary by species or tissue. We have observed an exacerbation of oxidative status in quiescent stromal cells treated with cortisol (30 and 300 ng/mL). It is worth revealing the regulation function of cortisol on the oxidation status in epithelial cells, which are the first line of defense against pathogen invasion.

The exposure of bovine endometrial cells to *Escherichia coli* LPS has been used to induce cellular oxidative injury [27,28]. The present study aimed to investigate the effect and mechanism of cortisol on the oxidative status of primary BEEC in response to LPS, and whether Se protected the cells from oxidative stress in the presence of high-level cortisol. Herein, we hypothesized that a high cortisol level has an influence on BEEC oxidative stress, and that Se supplementation enhances the antioxidant capacity of BEEC subjected to cortisol. We assessed the markers of oxidative stress, including ROS, MDA, and LDH, the antioxidant enzyme activities such as CAT, SOD, TRXR, and GSH-Px, and the activation of the Nrf2 pathway.

## 2. Materials and Methods

### 2.1. BEEC Isolation and Primary Cell Culture

The cells were isolated as described from previous reports [29]. Cattle from the experimental farm at Yangzhou University were sent to the local abattoir and were culled. Bovine uteri from post-pubertal nonpregnant cattle with no evidence of genital disease or microbial infection were collected and kept on ice until further processing in the laboratory. Briefly, the uterine horns were cut into pieces 3–4 cm long and the tissue was digested with 0.1% protease from *Streptomyces griseus* (P5147, Sigma, St. Louis, MO, USA) diluted in Dulbecco’s modified Eagle’s medium/F12 (DMEM/F-12) (D8900, Sigma, MO, USA) at 4 °C for 18 h. Then, the uterine horns were incised to facilitate scraping the endometrium. The scraped tissue was washed, collected, and centrifuged at 290× *g* for 5 min. The cells were resuspended in DMEM/F-12 containing 15% fetal bovine serum (FBS) (S711-001S, LONSERA, Suzhou, China) and 50 U/mL penicillin/streptomycin (S711-001S, Biosharp, Hefei, China). These cells were seeded in 25 cm^2^ flasks (TCF012050, Jet Biofil, Guangzhou, China) and cultured at 37 °C containing 5% CO_2_ in a cell culture incubator (HERAcell 150i, Thermo Fisher Scientific, Waltham, MA, USA). The medium was changed when the cells reached 90% confluence. The purification of BEEC was confirmed to be above 99% by detecting CK-18 using immunohistochemistry.

### 2.2. Experiment Design and Treatments

This study comprised three parts. In part one, we evaluated the effect of cortisol on the oxidative stress of BEEC induced by LPS. The cells were seeded onto 60 mm cell culture dishes (TCD010060, Jet Biofil, Guangzhou, China) at a density of 5 × 10^6^ cells per well. First, to observe the impact of cortisol alone on oxidative status, the cells were treated with a basal medium, or a basal medium containing various concentrations of cortisol (5, 15, and 30 ng/mL). The group arrangement was as follows: the blank control group, the cortisol treatment groups (5, 15, and 30 ng/mL). Then, in order to determine the influence of cortisol on BEEC oxidative stress, the cells were divided into five groups: the blank control group, the LPS treatment group (LPS group), the co-treatment groups of LPS and cortisol (5, 15, and 30 ng/mL). Specifically, the cells in the LPS group were cultured in the basal medium containing LPS to establish an oxidative stress model. In the other three groups, the cells were treated with the basal medium containing LPS and a series of concentrations of cortisol. LPS (L2880, Sigma, MO, USA) was dissolved into a 1 mg/mL solution with the basal culture medium and repacked for later use. The working concentration of LPS was 1 μg/mL [28]. Cortisol (H0888, Sigma, MO, USA) was dissolved in the basal medium and was diluted to 1 μg/mL as a stock solution. Concentrations of 5, 15, and 30 ng/mL cortisol were within the pathophysiological range and were selected in the present study [30].

Next, we observed the effect of Se on BEEC oxidative stress in the presence of cortisol. Sodium selenite (S5261, Sigma, MO, USA) was dissolved in the basal culture medium and stored at a concentration of 1 mM. Se concentrations of 1, 2, and 4 μM were selected by detecting the cell viability and the LDH levels during the preliminary trials (Figure S1). The Se status was confirmed by detecting the gene expression of glutathione peroxidase 1 (Figure S2 and Table S1). The effect of Se on the oxidative status of BEEC was first checked by the treatment of 1, 2, and 4 μM Se for 12 h. The groups were then marked as follows: the control group, the LPS group, the LPS plus cortisol group (LC), and the LPS, cortisol, plus Se (1, 2, and 4 μM) groups (LCS1, LCS2, or LCS4). The concentration of cortisol was 30 ng/mL. In the three LCS groups, the cells were pretreated with 1, 2, or 4 μM Se for 12 h.

To gain an insight into the mechanism of cortisol, the nonsteroidal glucocorticoid receptor antagonist AL 082D06 (D06) (HY-15709, MCE, Shanghai, China) was used. Based on the preliminary cell viability test (Figure S3) and the related reference [31], D06 was dissolved in DMSO to 1 mM for storage, and was further diluted in the basal culture medium to the concentration of 80 nM for use. After the pretreatment with 4 μM Se for 12 h, the cells were challenged with LPS, COR, and/or D06, and seven parallel groups were prepared: the control group, the LPS group, the LPS plus Se group (LPS + Se), the LPS plus cortisol group (LPS + COR), and the LPS, cortisol, plus Se group (LPS + COR + Se), the LPS, cortisol, plus D06 group (LPS + COR + D06), and the LPS, cortisol, Se, plus D06 group (LPS + COR + Se + D06).

### 2.3. Indicators of Oxidative Stress

#### 2.3.1. Intracellular ROS, LDH, and MDA

The intracellular ROS level was detected by a ROS detection kit (S0033S, Beyotime Biotechnology, Shanghai, China) with an oxidation-sensitive fluorescent probe (DCFH-DA). After being deacetylated by nonspecific esterase, DCFH-DA was converted to fluorescent dichloro-fluorescein (DCF) and was further oxidized by ROS. After treatment as previously described, cells were washed three times with phosphate-buffered saline (PBS), digested with trypsin, and collected. Then, the cells were loaded with DCFH-DA. DCFH-DA was diluted in the basal culture medium (dilution ratio 1:1000) and incubated with cells for 30–50 min at 37 °C according to the manufacturer’s instruction. DCF fluorescence was detected by a FACScan flow cytometer (CytoFLEX S, BECKMAN COULTER, Shanghai, China) within less than 1 h.

MDA (A003-4-1) and LDH (A020-2-2) levels in BEEC were assayed according to the instructions of the specific kit obtained from Jiancheng Bioengineering Institute (Nanjing, China). LDH was measured using the cell culture medium. MDA determination required protein extraction from the cells.

#### 2.3.2. Intracellular CAT, SOD, GSH-PX, and TRXR Activity and T-AOC

The cells were treated as described above. Different indicators were determined according to the instructions of the corresponding kits, which were obtained from Jiancheng Bioengineering Institute (A007-1-1 for CAT, A001-3 for SOD, A005-1-2 for GSH-PX, A119-1-1 for TRXR, and A015-2-1 for T-AOC). Before detection, protein extraction was required and the steps were as follows: the adherent cells were collected using a cell scraper and transferred to a centrifuge tube. PBS of 200 μL was added to the tube. An ultrasonic crusher was used to break the cells three times, with 10 s each time at a frequency of 65 Hz. Subsequently, the samples were centrifuged at 290× *g* at 4 °C for 10 min, and the supernatant was obtained to determine of the contents of the related antioxidant enzymes. The diluted supernatant was used to measure the protein concentration by a bicinchoninic acid assay kit (P0010, Beyotime Biotechnology, Shanghai, China).

### 2.4. RNA Extraction and Quantitative Real-Time Polymerase Chain Reaction (qPCR)

Total RNA was extracted from BEEC using Trizol (R401, Vazyme, Nanjing, China), according to the manufacturer’s instructions, and quantified using a Nanodrop 2000 spectrophotometer (Thermo Fisher Scientific, Wilmington, NC, USA). RNA samples with an A260/A280 ratio between 1.8 and 2.1 were selected for further analysis. The cDNA was synthesized from 1 μg of total RNA using *TransScript*^®^ Uni All-in-One First-Strand cDNA Synthesis SuperMix for qPCR (AU341-02, TransGen Biotech, Beijing, China). The RT-qPCR was conducted in a 20 μL reaction system according to the recommended conditions of *PerfectStart*^®^ Green qPCR SuperMix (AQ601-01-V2, TransGen Biotech, Beijing, China) using the CFX Connect™ Real-Time System (Bio-Rad Laboratories, Hercules, CA, USA). The primers were designed based on the sequence of *beta-actin* (*ACTB*), *nuclear factor erythroid 2-related factor 2* (*NFE2L2*), *heme oxygenase 1* (*HMOX1*), *NAD(P)H quinone dehydrogenase 1* (*NOQ1*), *glutathione peroxidase 1* (*GPX1*), and *glutathione peroxidase 4* (*GPX4*) genes of *Bos taurus* published in GenBank. The primer sequences used in this study are listed in Table 1. The 2^−ΔΔCt^ method was used to calculate the relative abundance of gene transcripts.

### 2.5. Protein Extraction and Western Blotting

Following treatment, the total protein was extracted. The cells were washed with PBS three times. Each dish (60 mm dish) was added with 200 μL RIPA lysis buffer (C1053, Applygen, Beijing, China) containing 2 μL protease inhibitors (P1265, Applygen, Beijing, China) and 2 μL phosphatase inhibitors (DP1260, Applygen, Beijing, China), and the cells were gently scraped off and transferred to a centrifuge tube. The cells were lysed for 10 min on ice. The samples were centrifuged at 290× *g* for 10 min at 4 °C, and the supernatant was collected. The protein concentration of each group was determined by the bicinchoninic acid method and the total protein concentration was adjusted by the mixture of RIPA lysate. After denaturation with a 4 × SDS loading buffer (P1015, Solarbio, Beijing, China) in a 99 °C water bath, the protein samples were collected for subsequent procedures, or stored at −80 °C until use. The samples were used within a week.

The protein expression of NRF2, heme oxygenase (HO-1), NADPH dehydrogenase (NQO1), GPX1, and GPX4 was determined using Western blot. Protein at around 20 μg was loaded into each gel well, and was separated by sodium dodecyl sulfate–polyacrylamide gel electrophoresis and transferred to polyvinylidene fluoride membranes (IPVH00010, Millipore, Darmstadt, Germany). Specifically, a 12% separation gel and an 8% concentration gel were prepared, and the samples were separated and concentrated under a 120 V condition for 60–90 min and 80 V condition for 30 min, respectively. The protein transfer condition was 200 mA for 60 min. Then, the membrane was blocked in 5% skim milk for 1 h. After washing with Tris-buffered saline containing Tween 20 (TBST) three times for 5 min each time, the membrane was incubated with the corresponding primary antibody overnight at 4 °C with shaking. The primary antibodies were NRF2 (AF0639, Affbiotech, Changzhou, Jiangsu, China), HO-1 (10701-1-AP, Proteintech Group, Wuhan, China), NQO1 (DF6437, Affbiotech, Jiangsu, China), GPX1 (29329-1-AP, Proteintech Group, Wuhan, China), GPX4 (sc-50497, Santa Cruz Biotechnology, Dallas, TX, USA), and β-actin (20536-1-AP, Proteintech Group, Wuhan, China). After the wash with TBST, the membranes were incubated with HRP-labeled secondary antibodies for 1 h at room temperature with shaking. Specifically, the goat anti-rabbit secondary antibody (S0001, Affbiotech, Jiangsu, China) was used for the primary antibody above. Again, the membrane was repeatedly washed with TBST. Finally, the blots were imaged using a ChemiScope5300Pro CCD camera (Clinx Science Instruments, Shanghai, China). The gray values of the blots were quantified by ImageJ software (9.0 version) (National Institutes of Health, Bethesda, MD, USA). The samples were randomized for blotting and were normalized to β-actin for quantitative assessment.

### 2.6. Immunofluorescence Staining

The cells were seeded at a density of 1 × 10^5^ cells per well into 24-well plates (TCP011024, Jet Biofil, Guangzhou, China) with preloaded glass coverslips (801010, NEST Scientific, Wuxi, China) and were treated as mentioned above. Then, the cells were washed with 4 °C precooled PBS three times for 5 min each time. The precooled 4% paraformaldehyde (BL539A, biosharp, Beijing, China) solution of 350 μL was added to each well for 15 min to fix the cells. Then, the membranes were permeabilized with 0.4% TritonX-100 (ST797, Beyotime, Shanghai, China) for 15 min at room temperature. After washing three times with PBS, 250 μL of 5% BSA (A8020, Solarbio, Beijing, China) was added to each well and incubated at 37 °C for 1–1.5 h. After washing, the slide was incubated with 250 μL diluted NRF2 antibody at 4 °C overnight. Then, 250 μL of the diluted FITC-conjugated secondary antibody (A0423, Beyotime, Shanghai, China) was added to each well for 1–1.5 h at room temperature in a dark room. From then on, attention was paid to avoid light throughout. The slides were washed with PBS three times for 5 min each time, and were stained with DAPI for 15 min at room temperature. Finally, after washing, the cells were transferred to slides, which were dripped with an anti-quenching solution in advance, sealed, and stored at 4 °C until observation. The cells were visualized using a fluorescence microscope (Leica TCS SP8 STED, Leica, Wetzlar, Germany).

### 2.7. Statistical Analysis

All experiments were repeated at least three times. The data were analyzed by GraphPad Prism 9.0 (Dotmatics, Boston, MA, USA) software. The Kolmogorov–Smirnov method was used to test the normal distribution of the data. All data followed a normal distribution (*p* > 0.05). Statistically significant differences were calculated by one-way ANOVA, followed by Dunnett’s or Tukey’s multiple comparisons test. The results were expressed as the means  ±  standard error of means (SEM). Values of *p* < 0.05 and *p* < 0.01 were considered statistically significant and extremely significant, respectively.

## 3. Results

### 3.1. Cortisol Enhanced the Antioxidant Capacity of BEEC

**Figure 1:**
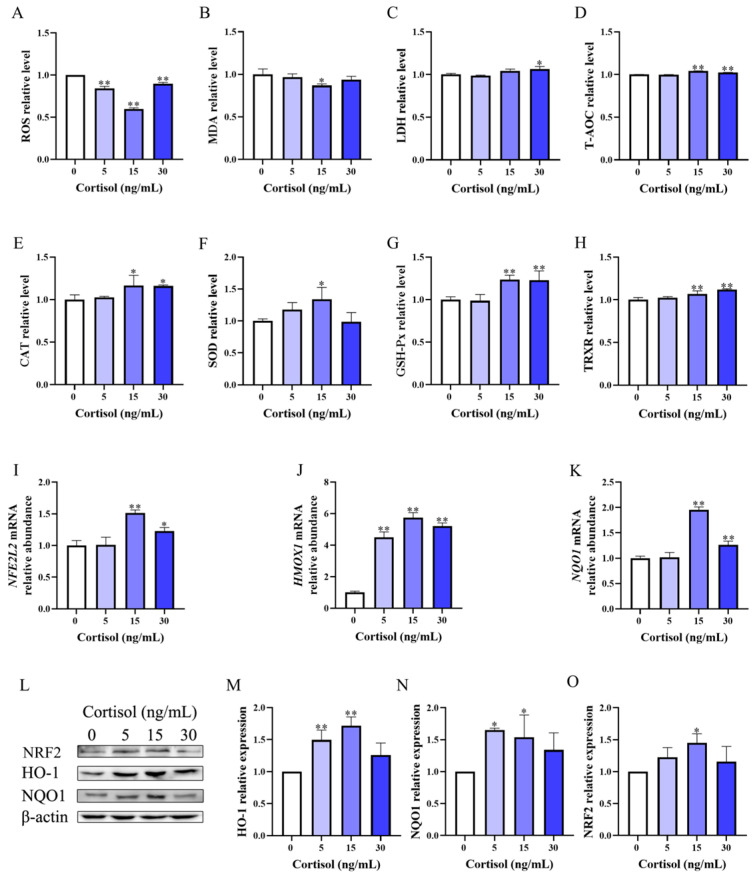
The effect of cortisol alone on the oxidative status of primary bovine endometrial epithelial cells. The cells were stimulated with 5, 15, and 30 ng/mL cortisol for 12 h to detect the changes in the relative level of reactive oxygen species (ROS, panel (**A**)), malondialdehyde (MDA, panel (**B**)), lactate dehydrogenase (LDH, panel (**C**)), total antioxidant capacity (T-AOC, panel (**D**)), catalase (CAT, panel (**E**)), superoxide dismutase (SOD, panel (**F**)), glutathione peroxidase (GSH-Px, panel (**G**)), and thioredoxin reductase (TRXR, panel (**H**)). The key mRNA (after 12 h treatment) and protein (after 90 min treatment) expression of the Nrf2/HO-1 pathway was determined and quantified using qPCR and Western blot (**I**–**O**). The β-actin was used as the internal control. The data are presented as means ± SEM (*n* = 3). * *p* < 0.05, ** *p* < 0.01 vs. the control group. The samples derived from the same experiment and the blots were processed in parallel.

The changes in oxidative markers of BEEC treated with cortisol alone are shown in Figure 1A–H. Generally, cortisol resulted in a decrease in ROS (*p* < 0.01) and MDA (*p* < 0.05) levels, with the lowest level observed in the 15 ng/mL cortisol intervention group. Cortisol had no effect (*p* > 0.05) on LDH in the 5 and 15 ng/mL treatment groups, and caused a slight increase in the LDH level in the 30 ng/mL treatment group (*p* < 0.05). There was an increase in the contents of T-AOC (*p* < 0.01), CAT (*p* < 0.05), GSH-Px (*p* < 0.01), and TRXR (*p* < 0.01) after 15 and 30 ng/mL cortisol treatment, and SOD activity (*p* < 0.05) in cells treated with 15 ng/mL cortisol. No change (*p* > 0.05) was found in these oxidative markers in BEEC of the 5 ng/mL cortisol group, except ROS.

The changes in the relative abundance of the Nrf2 pathway-related gene transcripts and proteins are demonstrated in Figure 1I–O. Cortisol of 15 and 30 ng/mL caused increased (*p* < 0.05) relative mRNA abundance of *NFE2L2*, *HMOX1*, and *NQO1*, with the 15 ng/mL cortisol group showing the greatest change (1.5-, 5.4-, and 2.0-fold, respectively). Comparatively, the 5 ng/mL cortisol treatment only resulted in a 4.5-fold increase (*p* < 0.05) in the relative abundance of *HMOX1* mRNA. The elevation in the protein levels of NRF2 (*p* < 0.05), HO-1 (*p* < 0.01), and NQO1 (*p* < 0.05) was most prominent in BEEC treated with 15 ng/mL cortisol, followed by the 5 ng/mL cortisol group. Cortisol of 30 ng/mL did not upregulate these protein levels with statistical significance. Collectively, there was an increased antioxidant capacity of BEEC treated with various concentrations of cortisol, in which 15 ng/mL cortisol evoked the most prominent effect.

### 3.2. Cortisol Alleviated LPS-Induced Oxidative Stress

**Figure 2:**
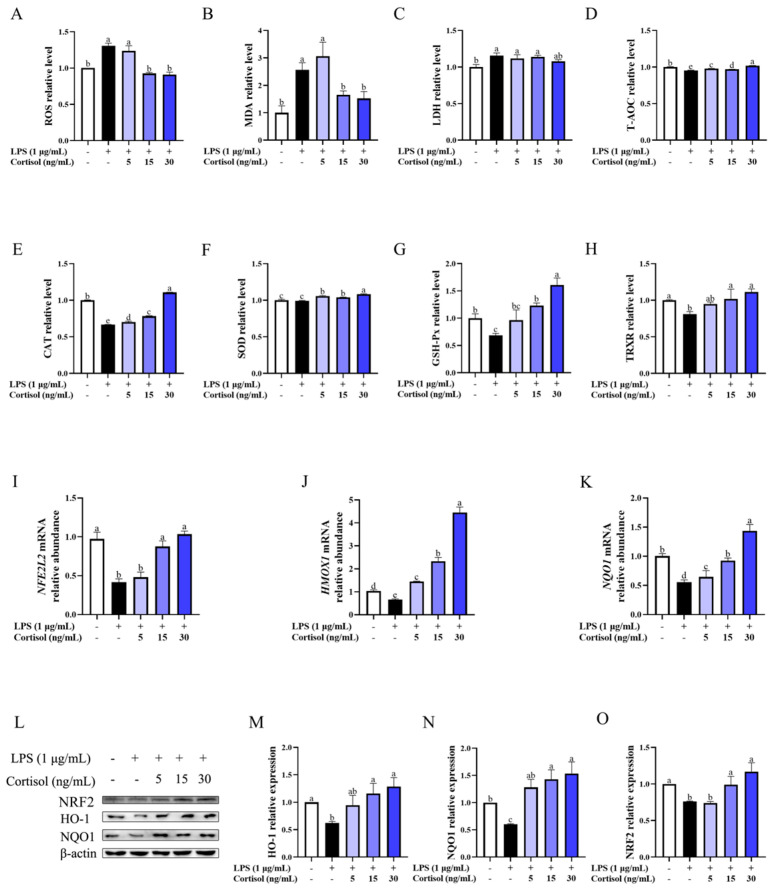
The effect of cortisol (5, 15, and 30 ng/mL) on the oxidative injury of primary bovine endometrial epithelial cells. The oxidative stress model was established by 1 μg/mL lipopolysaccharide (LPS) stimulation. The cells were co-treated with cortisol and LPS for 12 h to detect the changes in reactive oxygen species (ROS, panel (**A**)), malondialdehyde (MDA, panel (**B**)), lactate dehydrogenase (LDH, panel (**C**)), total antioxidant capacity (T-AOC, panel (**D**)), catalase (CAT, panel (**E**)), superoxide dismutase (SOD, panel (**F**)), glutathione peroxidase (GSH-Px, panel (**G**)), and thioredoxin reductase (TRXR, panel (**H**)). Furthermore, the relative abundance *NFE2L2*, *HMOX1*, and *NQO1* mRNA (**I**–**K**) was determined after 12 h treatment by qPCR. The total protein expression of NRF2, HO-1, and NQO1 (**L**–**O**) was determined after 90 min treatment by Western blot. The β-actin protein was used as the internal control. The data are presented as the means ± SEM (*n* = 3). Different letters indicate statistical difference (*p* < 0.05), while the same letter indicates no statistical difference. The samples derived from the same experiment and the blots were processed in parallel.

Figure 2 depicts the influence of cortisol on the indicated oxidative markers of BEEC challenged with LPS. In general, LPS of 1 μg/mL induced the production (*p* < 0.05) of ROS, MDA, and LDH by about 1.2- to 2.5-fold. There was a decrease in the contents of CAT (*p* < 0.05), GSH-Px (*p* < 0.05), TRXR (*p* < 0.05), and T-AOC (*p* < 0.05), and in the relative abundance of Nrf2, HO-1, and NQO1 at mRNA and protein (*p* < 0.05) levels in response to LPS stimulation.

Compared with the LPS group, cortisol of 15 and 30 ng/mL enhanced the antioxidant defense of BEEC, as observed by the decrease (*p* < 0.05) in ROS and MDA levels, the increase in the contents of CAT (*p* < 0.05), SOD (*p* < 0.05), GSH-Px (*p* < 0.05), and TRXR (*p* < 0.05). Meanwhile, there was an elevation (*p* < 0.05) in the relative abundance of *NFE2L2*, *HMOX1*, and *NQO1* gene transcripts and the corresponding protein levels in BEEC co-treated with LPS and cortisol (15 and 30 ng/mL) compared with the cells in the LPS group. Such changes were affected to a lesser extent by 5 ng/mL cortisol than cortisol of 15 and 30 ng/mL. Generally, the variation tendency of the Nrf2/HO-1 pathway at mRNA and protein levels was consistent with the change in oxidative indicators and enzyme contents caused by cortisol, and cortisol of 30 ng/mL most effectively alleviated the LPS-induced oxidative stress.

### 3.3. Se Alone Enhanced the Antioxidant Capacity of BEEC

**Figure 3:**
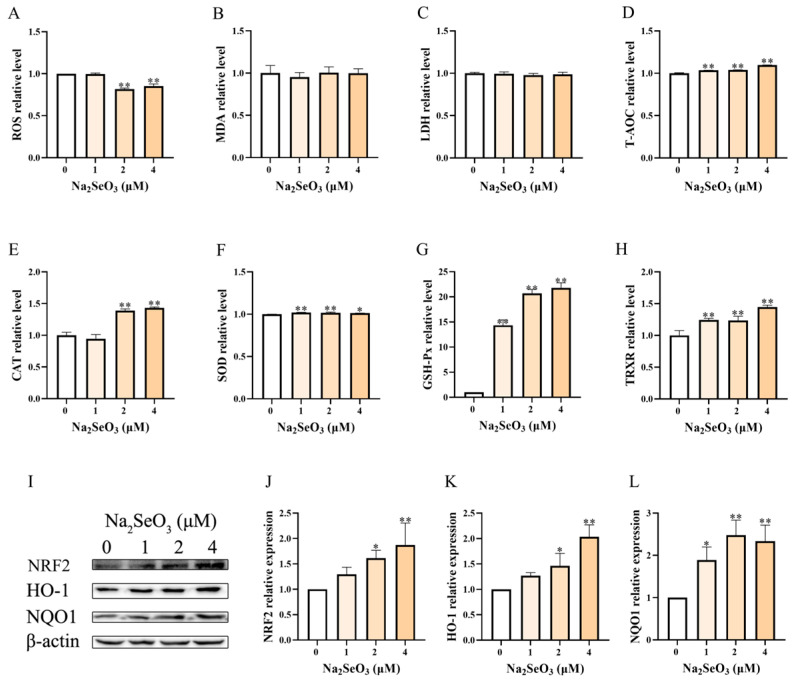
The effect of selenium (Se) alone on the oxidative status of primary bovine endometrial epithelial cells. The cells were pretreated with 1, 2, and 4 μM sodium selenite for 12 h. The cells were then treated for an additional 12 h to detect the changes in reactive oxygen species (ROS, panel (**A**)), malondialdehyde (MDA, panel (**B**)), lactate dehydrogenase (LDH, panel (**C**)), total antioxidant capacity (T-AOC, panel (**D**)), catalase (CAT, panel (**E**)), superoxide dismutase (SOD, panel (**F**)), glutathione peroxidase (GSH-Px, panel (**G**)), and thioredoxin reductase (TRXR, panel (**H**)), and to determine the total protein expression of the Nrf2/HO-1 pathway using Western blot (**I**–**L**). The β-actin was used as the internal control. The data are presented as means ± SEM (*n* = 3). * *p* < 0.05, ** *p* < 0.01 vs. the LPS group. The samples derived from the same experiment and the blots were processed in parallel.

As shown in Figure 3A–H, the basal antioxidant capacity of BEEC was enhanced by Se of 2 and 4 μM, with a moderate decrease (*p* < 0.01) in ROS production and various degrees of increase in the contents of T-AOC (around 1-fold, *p* < 0.01,), CAT (1.4-fold, *p* < 0.01), SOD (1-fold, *p* < 0.05), GSH-Px (15- to 22-fold, *p* < 0.01), and TRXR (1.2- to 1.4-fold, *p* < 0.01). Except for the elevated levels of T-AOC, GSH-Px, and TRXR, Se of 1 μM showed no impact (*p* > 0.05) on these oxidative indicators. The MDA and LDH were unaffected (*p* > 0.05) by Se. The levels in T-AOC, CAT, GSH-Px, and TRXR seemed to increase with the elevation of Se concentration.

This changing trend was also evident in the key protein levels of the Nrf2/HO-1 pathway (Figure 3I–L). Specifically, the elevations of NRF2 (1.5- to 2.3-fold, *p* < 0.01), HO-1 (1.7- to 2.2-fold, *p* < 0.01), and NQO1 (2.0- to 2.7-fold, *p* < 0.01) protein were most prominent in the 4 μM Se group, followed by the 2 μM Se group (*p* < 0.05). One μM Se also increased these protein levels, but only NQO1 showed statistical significance. Generally, Se (1, 2, and 4 μM) alone optimized the basal antioxidant status of BEEC alone with the Nrf2 activation in a dose-dependent manner.

### 3.4. Se Alleviated LPS-Induced Oxidative Stress of BEEC in the Presence of Cortisol

**Figure 4:**
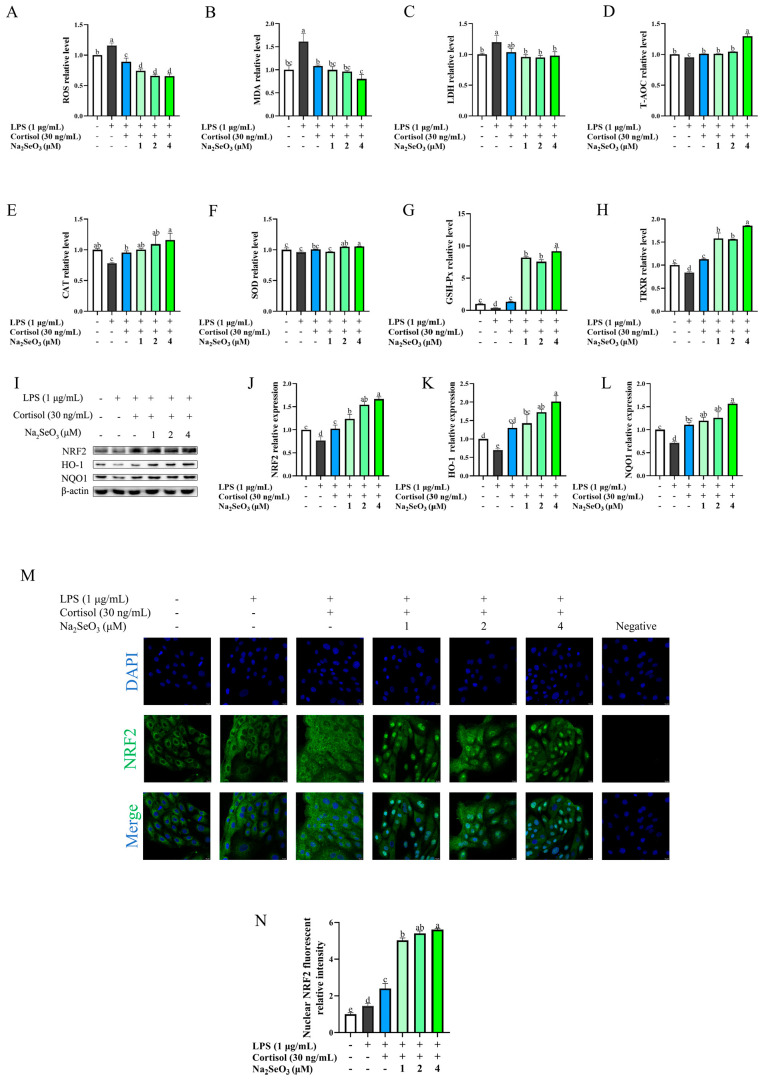
The effect of selenium (Se) on the oxidative stress of primary bovine endometrial epithelial cells under a high level of cortisol. The cells were pretreated with 1, 2, and 4 μM sodium selenite for 12 h. Then, the cells were co-treated with cortisol (30 ng/mL) and lipopolysaccharide (LPS, 1 μg/mL) to measure the related antioxidant makers, including reactive oxygen species (ROS, panel (**A**)), malondialdehyde (MDA, panel (**B**)), lactate dehydrogenase (LDH, panel (**C**)), total antioxidant capacity (T-AOC, panel (**D**)), catalase (CAT, panel (**E**)), superoxide dismutase (SOD, panel (**F**)), glutathione peroxidase (GSH-Px, panel (**G**)), and thioredoxin reductase (TRXR, panel (**H**)), and to detect the total protein level of NRF2, HO-1, and NQO1 (**I**–**L**) using Western blot. The localization of NRF2 protein was detected by immunofluorescence (**M**,**N**). The β-actin protein was used as the internal control. The data are presented as the means ± SEM (*n* = 3). Different letters indicate statistical difference (*p* < 0.05), while the same letter indicates no statistical difference. The samples derived from the same experiment and the blots were processed in parallel.

In the context of high cortisol levels, we investigated whether there would be any change in Se antioxidation (Figure 4). The impact of cortisol on the oxidative markers and the NRF2 pathway in LPS-stimulated BEEC was similar to those described above (Figure 4, blue panel). On the basis of LPS and cortisol treatment, preincubation with Se (1, 2, and 4 μM) further reduced the levels of ROS (*p* < 0.01) and increased the contents of GSH-Px (*p* < 0.01) and TRXR (*p* < 0.01). The magnitude of change in MDA, T-AOC, CAT, and SOD was minimal (*p* > 0.05) in response to 1 and 2 μM Se, and became greater (*p* < 0.01) in response to 4 μM Se compared with the LC group. LDH was unaffected (*p* > 0.05) by Se. Compared with the LC group, Se seemed to promote (*p* < 0.05) the protein level of NRF2, HO-1, and NQO1 in a dose-dependent manner (Figure 4I–L).

NRF2 localization in response to cortisol and Se was visualized by the immunofluorescence assay (Figure 4M,N). In the control group, NRF2 protein was more distributed in the cytoplasm than that in the nucleus. The nuclear fluorescence intensity of NRF2 was mildly increased (*p* < 0.05) by LPS stimulation, and became obvious (*p* < 0.01) after the addition of cortisol. This already elevated level of nuclear NRF2 was further enhanced (*p* < 0.01) by Se pretreatment (1, 2, and 4 μM), at which time the fluorescence intensity in the nucleus was obviously higher than that in the cytoplasm.

### 3.5. Cortisol Antioxidation Was Mediated by the Cortisol Receptor

**Figure 5:**
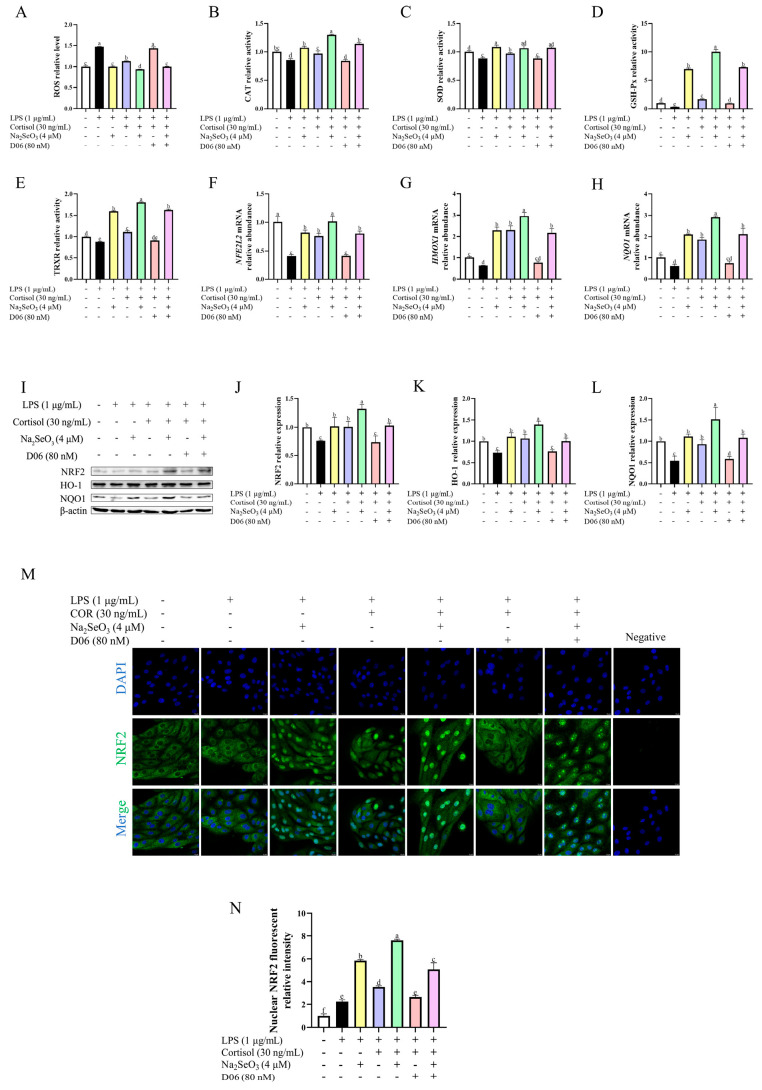
The antioxidative effect of cortisol was abolished by blockage of the cortisol receptor in primary bovine endometrial epithelial cells. The cells were pretreated with 4μM sodium selenite for 12 h. Then, the cells were co-treated with lipopolysaccharide (LPS, 1 μg/mL), cortisol (COR, 30 ng/mL), and AL 082D06 (D06, 80 nM) for 12 h to measure the related antioxidant makers, including reactive oxygen species (ROS, panel (**A**)), catalase (CAT, panel (**B**)), superoxide dismutase (SOD, panel (**C**)), glutathione peroxidase (GSH-Px, panel (**D**)), and thioredoxin reductase (TRXR, panel (**E**)), and to detect the key mRNA and the total protein level of the Nrf2/HO-1 pathway (**F**–**L**) using qPCR and Western blot. Localization of the NRF2 protein was detected after 90 min treatment by immunofluorescence (**M**,**N**). The β-actin protein was used as the internal control. The data are presented as the means ± SEM (*n* = 3). Different letters indicate statistical difference (*p* < 0.05), while the same letter indicates no statistical difference. The samples derived from the same experiment and the blots were processed in parallel.

Since cortisol elicited an antioxidative effect, we examined whether blockage of the cortisol receptor, by using D06, had any effect on its antioxidative property (Figure 5). Compared with the LPS + COR group (blue panel), the addition of D06 (red panel) resulted in an increased (*p* < 0.01) ROS level and the decreased (*p* < 0.05) contents of antioxidant enzymes, along with the decreased abundance of Nrf2, HO-1, and NQO1 at mRNA (*p* < 0.01) and protein (*p* < 0.01) levels. In the presence of Se, similar results were obtained when we made comparisons between the LPS + COR + Se group (green panel) and the LPS + COR + Se + D06 group (pink panel), where the already enhanced antioxidant capacity and Nrf2 activation was partially blunted by blockage of the cortisol receptor. Moreover, a D06-inhibitable increase in the nuclear NRF2 fluorescence intensity was observed in cortisol-treated cells, and this was consistent with the results mentioned above, suggesting the involvement of the cortisol receptor in cortisol antioxidation.

### 3.6. Cortisol Affected GPX1 and GPX4 Expression in the Presence of Se

**Figure 6:**
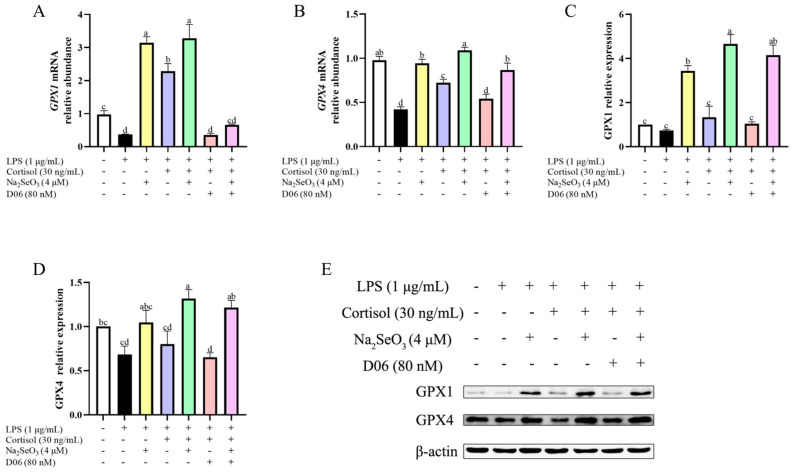
The changes in glutathione peroxidase 1 (GPX1) and glutathione peroxidase 4 (GPX4) expressions in lipopolysaccharide (LPS)-stimulated primary bovine endometrial epithelial cells treated with cortisol and selenium. The cells were pretreated with 4 μM sodium selenite for 12 h. Then, the cells were co-treated with cortisol (30 ng/mL), AL 082D06 (D06, 80 nM), and LPS (1 μg/mL) to detect the changes in the relative abundance of *GPX1* and *GPX4* in mRNA (**A**,**B**) and the corresponding protein levels (**C**–**E**) using qPCR and Western blot, respectively. The β-actin protein was used as the internal control. The data are presented as the means ± SEM (*n* = 3). Different letters indicate statistical difference (*p* < 0.05), while the same letter indicates no statistical difference. The samples derived from the same experiment and the blots were processed in parallel.

Based on the obtained result that cortisol caused the increased contents of GSH-Px, we determined to detect the changes in glutathione peroxidase 1 (GPX1) and glutathione peroxidase 4 (GPX4). As shown in Figure 6, the relative abundance of gene transcripts and proteins of GPX1 and GPX4 was generally decreased by LPS, whereas there was a marked increase (all *p* < 0.01) in these indicators with Se supplementation regardless of the presence of cortisol or D06. Although the mRNA abundance of *GPX1* and *GPX4* was higher in the LPS + COR group (blue panel) than in the LPS group (black panel), there was no difference in the corresponding protein levels between these two groups. However, in the presence of Se, cortisol caused a mild increase (all *p* < 0.01) in GPX1 and GPX4 at both mRNA and protein levels (LPS + Se group vs. LPS + Se + COR group), which can be weakened by blockage of the cortisol receptor (LPS + Se + COR group vs. LPS + Se + COR + D06 group). Collectively, cortisol may affect the expression of GPX1 and GPX4 in the presence of Se.

## 4. Discussion

This experiment found that cortisol, within the physiological concentration range, promoted antioxidant capacity and Nrf2 activation in BEEC with or without LPS. The observed Se antioxidation was accompanied by elevated contents of antioxidant enzymes and Nrf2/HO-1 activation, and was further enhanced by cortisol. This cortisol-induced enhancement was abolished by a glucocorticoid receptor antagonist.

Normally, the cortisol level of dairy cows is approximately 5 ng/mL before parturition, and rises sharply during delivery, reaching 19.2 ng/mL [32]. Based on the actual physiological concentration of cows that has been confirmed [33], cortisol concentrations of 5, 15, and 30 ng/mL were selected in this experiment. Our results showed that 5, 15, and 30 ng/mL of cortisol generally enhanced the antioxidant effect of BEEC to a varying degree. Specifically, when cortisol was 15 ng/mL, the upregulated contents of antioxidant enzymes were most prominent. The relationship between oxidative stress and cortisol levels has been seldom studied in BEEC. There have been in vitro and in vivo studies examining the changes in cortisol and oxidative indices in bovine blood or saliva exposed to various stressors [34,35,36]. However, due to the difference in experiment design, treatment methods, and the reported results, it is difficult to relate these observations to the current research. To verify this observation, we expended the concentration range of cortisol to 1000 ng/mL, and obtained a similar effect to that of cortisol of 15 and 30 ng/mL (Figure S4). Our previous study found an opposite result in stromal cells, in which cortisol alone induced the oxidative stress of BESC [6]. The remarkable difference of cortisol on oxidative status between BEEC and BESC indicated that the effects of cortisol antioxidation varied among cell types.

In quiescent conditions, the NRF2 protein undergoes a constant degradation by binding to its negative regulator in the cytosol. Upon oxidative stress, the NRF2 degradation mechanism ceases, which results in NRF2 stabilization, accumulation, and nuclear translocation, where NRF2 binds to the antioxidant response element for the robust induction of cytoprotective genes encoding antioxidant enzymes [37]. Based on the obtained results that cortisol promoted Nrf2/HO-1 activation and improved the cellular antioxidant status, we assume that cortisol antioxidation is mediated by Nrf2 in BEEC. Interestingly in stromal cells, the effect of cortisol on the oxidative status of BESC was not accompanied by any change in Nrf2 pathway [6]. Furthermore, it has been demonstrated that exogenous corticosterone induced Nrf2 overexpression in melanoma cells [38], whereas glucocorticoids of 100 nM (36.247 ng/mL) impaired the Nrf2-dependent cellular antioxidant response in HEK-293 cells and hepatic H4IIE cells [39]. We speculate that the mechanism of cortisol modulating the cellular oxidative status is probably cell-specific.

LPS can induce oxidative stress in a variety of cells and has been reported in studies of endometrial cells in dairy cows [40]. In this study, 1 μg/mL LPS caused the production of ROS and MDA, suggesting the establishment of the oxidative stress model in BEEC. Here, we found that, in the presence of LPS, the antioxidant effect of cortisol was not evident at 5 ng/mL, but became pronounced when it reached 15 ng/mL, and was most obvious at 30 ng/mL. It is worth noting that the most evident concentration of cortisol showing antioxidant property was 15 ng/mL in resting state, and was 30 ng/mL in the state of oxidative stress. These results indicate that cortisol exhibited antioxidant effects in both conditions, but the mechanisms may be different. The oxidative stress caused by LPS seemed to upregulate the upper limit of cortisol level that produced an antioxidant effect. A team found that LPS induced the secretion of corticosterone to counteract the subsequent hypoxic stress in neonatal rats and explained that LPS induced an endogenous secretion of corticosterone and the inhibition of glucocorticoid receptors shifted the LPS response from preconditioning to sensitization [41]. Hence, our results may suggest that cortisol induces the establishment of endotoxin tolerance. It prepares the cells for the challenge and thereby prevents exaggerated oxidative injury. In addition, this trend of antioxidant effect corresponds to the anti-inflammatory effect of cortisol by the fact that cortisol (5, 15, and 30 ng/mL) inhibited the LPS-induced inflammation of BEEC in a dose-dependent manner [29], and that there is a positive correlation between the proinflammatory factors and the oxidative intermediates. Here, we observed that the Nrf2 pathway was activated by cortisol, whereas this activation was abolished by the use of cortisol receptor antagonist D06, confirming the role of Nrf2 in cortisol antioxidation. The functional cross-talk between the Nrf2 pathway and the classical inflammatory pathway nuclear factor-κB (NF-κB) has been confirmed [42], and whether cortisol exerts anti-inflammation and antioxidative activities through the interaction of the two pathways requires further investigation.

Se demonstrates a biphasic dose–response pattern, exhibiting toxic effects at high doses while possessing beneficial properties at very low doses [43]. In our treatment, the Se concentration in the basal medium was 45.63 μg/L [44], which was under the lower limit of the appropriate Se level (51 to 85 μg/L) of dairy cows [45]. The working concentrations of Se in LCS1, LCS2, and LCS4 groups have reached Se abundant levels (>100 μg/L) [46]. A study has revealed that supplementing cows with selenium yeast during the late stages of gestation, when the whole blood Se concentration is 371 ± 7 μg/L, enhances their postpartum antioxidant capacity without adverse effects [12]. Se treatment led to a decrease in intracellular levels of ROS and MDA, while simultaneously enhancing the activities of SOD, CAT, and GPX in BMEC and BESC [6,47]. These are consistent with our results, in which Se pretreatment reduced intracellular ROS levels, along with elevated levels of antioxidant enzymes (CAT, SOD, GSH-Px, and TRXR). GSH-Px and TRXR are exemplary selenoproteins that have redox activity and can catalyze the reduction in hydroperoxides by thiols [48]. Se-dependent GSH-Px exists in several forms and is widely and abundantly distributed in the body, where GPX1 utilizes glutathione to reduce hydroperoxides and is highly sensitive to Se levels, and GPX4 is found to be the only isoform that decreases phosphatidylcholine hydroperoxide [48,49]. The TRXR system functions to reduce the oxidoreductase thioredoxin in a NADPH dependent manner, and plays an important role in regulating the cellular redox balance [50]. The Se-enriched diet has been reported to exert a GPX1 expression–dependent impact on the related gene expression in mice [51]. The addition of sodium selenite resulted in the increased mRNA expression of GPX1 rather than GPX4 in the bovine mammary cell line MAC-T [52]. According to our result, the magnitude of increase in GSH-Px and TRXR was greater than other antioxidant enzymes, and there was a remarkable increase in GPX1 and GPX4 at mRNA and protein levels by Se supplement, suggesting the importance of GSH-Px and TRXR in scavenging ROS in BEEC. The appropriate Se supplement has been shown to alleviate oxidative stress through the NRF2 pathway in hepatocytes [53], dopaminergic cells [54], and the bovine endometrial cell line [17]. Similarly, we observed Nrf2 activation in Se-treated BEEC. These results imply that Se protects BEEC through the Nrf2 pathway and the antioxidant enzymes.

Then, we focused on effects of Se on LPS-induced oxidative damage in BEEC under the condition of 30 ng/mL of cortisol. In the presence of cortisol, Se enhanced the antioxidant biomarkers and Nrf2 activation, which was verified by the immunofluorescence result. This is in line with our previous observations in stromal cells, that a Se supplement has a more significant protective effect on BESC oxidative stress at high cortisol levels [6]. In addition, Se supplementation with the presence of cortisol has been reported to inhibit the LPS-induced inflammation more effectively than Se alone in BEEC [55], and whether this potent anti-inflammatory effect correlates to the observed antioxidation result requires further investigation. The Nrf2 abundance was inhibited by D06 in the LPS + Se + COR + D06 group to a level that approximated the LPS + Se group, but was still higher than in the LPS group, suggesting that Se antioxidation is related to but not limited to the Nrf2 pathway. Moreover, although the antioxidation mechanisms of both cortisol and Se involve Nrf2, their specific mechanism of action seems different. Subsequent research can investigate these differences by focusing on the key modulators of the Nrf2 pathway. To explore the influence of cortisol on selenoproteins, we selected to detect the GPX1 and GPX4 expression. The GPX1 and GPX4 protein levels were not affected by cortisol in the absence of Se, but were slightly increased by cortisol with the presence of Se. The effect of cortisol on selenoprotein expression is less commonly reported. It has been found that hydrocortisone (1 μg/mL) caused an increase of about 55% in GPX1 mRNA expression in the bovine mammary cell receiving 100 nmol/L sodium selenite [52], and that cortisol (30 ng/mL) elevated the expression of GPX1 and GPX4 mRNA in BESC pretreated with sodium selenite (2 or 4 μM) [44]. However, none of these studies detected GPXs at the protein level. There seems to be substantial space for research concerning the regulating mechanism of cortisol on GPXs and potentially other selenoproteins. Finally, in the current study, there is a lack of relevant research to verify the observed results using the gene silencing technique, or to testify the in vitro observations by conducting animal experiments on dairy cows. These will be elucidated in our future study.

## 5. Conclusions

In conclusion, 15 and 30 ng/mL of cortisol exert protective effects on the oxidative status of BEEC through the Nrf2 pathway. The cytoprotective effect of Se is attributed to the increased antioxidant enzyme activities and Nrf2 activation. The potency and efficacy of Se in protecting BEEC is independent of LPS, and can be enhanced by a high level of cortisol (30 ng/mL).

## Figures and Tables

**Table 1:** The primer sequences.

Gene	Primer Sequence (5′ → 3′)	Length (bp)	NCBI Accession
*ACTB*	F: CATCACCATCGGCAATGAGC	156	NM_173979.3
	R: AGCACCGTGTTGGCGTAGAG		
*NFE2L2*	F: CCCAGTCTTCACTGCTCCTC	165	NM_001011678.2
	R: TCAGCCAGCTTGTCATTTTG		
*HMOX1*	F: GGCAGCAAGGTGCAAGA	221	NM_001014912.1
	R: GAAGGAAGCCAGCCAAGAG		
*NQO1*	F: AACCAACAGACCAGCCAATC	154	NM_001034535.1
	R: CACAGTGACCTCCCATCCTT		
*GPX1*	F: CTTGCTGCTTGGCGGTCA	139	NM_174076.3R
	R: AGGGGAGGCTGGGATGGAT		
*GPX4*	F: CACCGCCGAGATGAGCTTTA	198	NM_174770.4
	R: ACGTGGCCCCGGTACTT		

## Data Availability

The data presented in this study are available in the article.

## Supplementary Materials

The following supporting information can be downloaded at: https://www.mdpi.com/article/10.3390/ani15081075/s1, Figure S1. The effect of different concentrations of Se (1–64 μM) on the cell viability (A) and the LDH release (B) in the primary bovine endometrial epithelial cells. LDH, lactate dehydrogenase. Data were presented as means ± SEM (*n* = 6). ** *p* < 0.01 vs. the control group. Figure S2. Se supplementation caused increased relative abundance of *GPX1* mRNA in primary bovine endometrial epithelial cells. The cells were treated with basal medium containing various concentrations of sodium selenite. The mRNA expression was detected by qPCR and was analyzed by the 2^−ΔΔct^ method. The primer sequences for *GPX1* detection were listed below. The data were presented as the means ± SEM (*n* = 3). *GPX1*, glutathione peroxidase 1. * *p* < 0.05 and ** *p* < 0. 01, vs. the control group. Figure S3. The effect of different concentrations of D06 (20–200 nM) on the cell viability in primary bovine endometrial epithelial cells. D06 showed no influence on the cell viability. Data were presented as means ± SEM (*n* = 3). Figure S4. The effect of different concentrations of cortisol (0~1000 ng/mL) on the ROS level in primary bovine endometrial epithelial cells. Data were presented as means ± SEM (*n* = 3). ROS, reactive oxygen species. ** *p* < 0.01 vs. the control group. Table S1. Primer sequences used for the mRNA detection.

## Abbreviations

The following abbreviations are used in this manuscript:

*ACTB*	Beta-actin
BEEC	bovine endometrial epithelial cells
BMEC	bovine mammary epithelial cell
BESC	bovine endometrial stromal cells
CAT	catalase
D06	AL 082D06
GPX1	glutathione peroxidase 1
GPX4	glutathione peroxidase 4
GSH-Px	glutathione peroxidase
HO-1	heme oxygenase
LDH	lactate dehydrogenase
LPS	lipopolysaccharide
MDA	malondialdehyde
*NFE2L2*	*Nuclear factor erythroid 2-related factor 2*
NO	reactive nitrogen
Nrf2	nuclear factor erythroid 2-related factor 2
NQO1	NAD(P)H quinone dehydrogenase 1
ROS	reactive oxygen species
Se	selenium
SEM	standard error of means
SOD	superoxide dismutase
T-AOC	total antioxidant capacity
TRXR	thioredoxin reductase
