# Supplementary material for: The Antioxidant Effect of Selenium Is Enhanced by Cortisol Through Nrf2 Pathway in Bovine Endometrial Epithelial Cells
Source: Animals (Basel). 2025 Apr 8;15(8):1075. doi: 10.3390/ani15081075 (PMC12024080; doi:10.3390/ani15081075)
Supplement: Supplementary file 1 [file animals-15-01075-s001.zip › Figure S2 and Table S1.pdf]

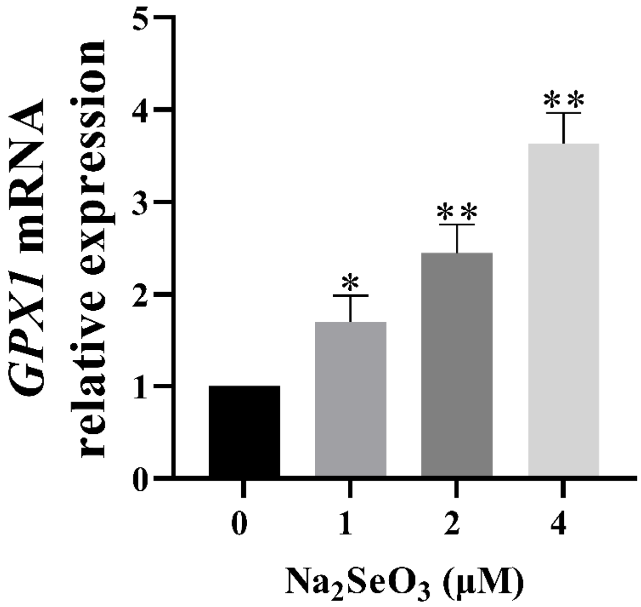

Figure S2. Se supplementation caused increased relative abundance of *GPX1* mRNA in primary bovine endometrial epithelial cells. The cells were treated with basal medium containing various concentrations of sodium selenite. The mRNA expression was detected by qPCR and was analyzed by the $2^{-\Delta\Delta ct}$ method. The primer sequences for *GPX1* detection were listed below. The data were presented as the means ± SEM (n = 3). *GPX1*, glutathione peroxidase 1. *$p < 0.05$ and **$p < 0.01$, vs. the control group.

Table S1. Primer sequences used for the mRNA detection

Gene	Primer sequences (5'→3')	Accession number
GPX1	F: CTTGCTGCTTGGCGGTCA<br>R: AGGGGAGGCTGGGATGGAT	NM_174076.3R
β-actin	F: CATCACCATCGGCAATGAGC	NM_173979.3

R: AGCACCGTGTTGGCGTAGAG
