# Supplementary material for: The Antioxidant Effect of Selenium Is Enhanced by Cortisol Through Nrf2 Pathway in Bovine Endometrial Epithelial Cells
Source: Animals (Basel). 2025 Apr 8;15(8):1075. doi: 10.3390/ani15081075 (PMC12024080; doi:10.3390/ani15081075)
Supplement: Supplementary file 1 [file animals-15-01075-s001.zip › Figure S3.pdf]

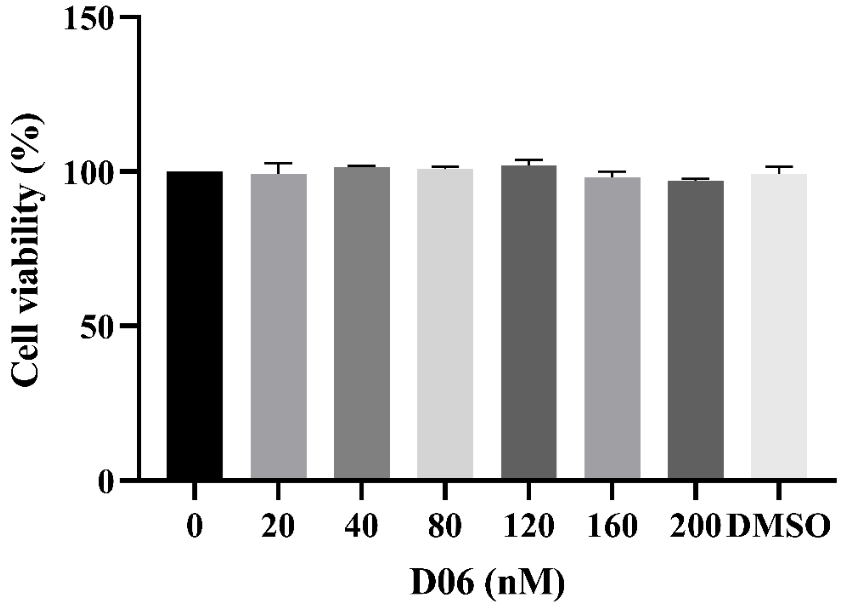

Figure S3. The effect of different concentrations of D06 (20-200 nM) on the cell viability in primary bovine endometrial epithelial cells. D06 showed no influence on the cell viability. Data were presented as means ± SEM (n = 3).
