# Supplementary material for: The Antioxidant Effect of Selenium Is Enhanced by Cortisol Through Nrf2 Pathway in Bovine Endometrial Epithelial Cells
Source: Animals (Basel). 2025 Apr 8;15(8):1075. doi: 10.3390/ani15081075 (PMC12024080; doi:10.3390/ani15081075)
Supplement: Supplementary file 1 [file animals-15-01075-s001.zip › Figure S4.pdf]

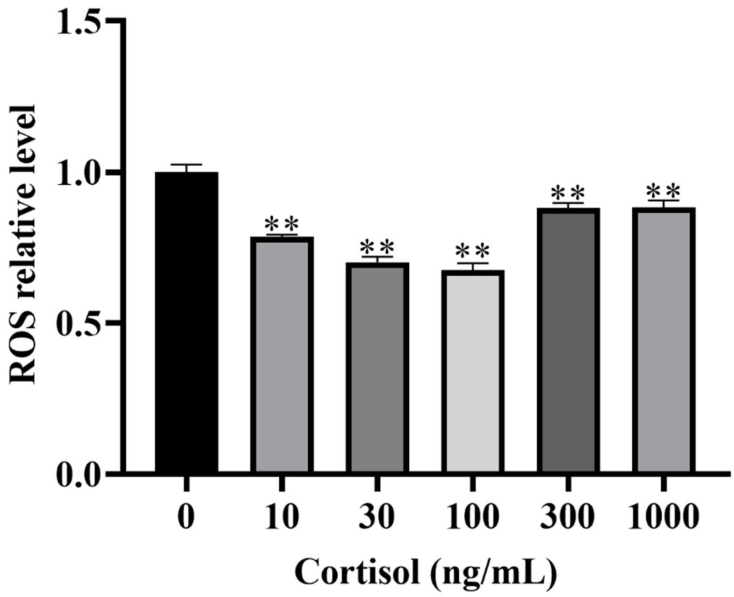

Figure S4. The effect of different concentrations of cortisol (0~1000 ng/mL) on the ROS level in primary bovine endometrial epithelial cells. Data were presented as means ± SEM (n = 3). ROS, reactive oxygen species. $^{**}p < 0.01$ vs. the control group.
